# Bis(4-hy­droxy­phen­yl) 1,4-phenyl­enebiscarbamate

**DOI:** 10.1107/S2414314622009191

**Published:** 2022-09-27

**Authors:** Isabel Martínez-de la Luz, Delia López-Velázquez, Sylvain Bernès, Jenaro L. Varela Caselis

**Affiliations:** a Facultad de Ciencias Químicas, Benemérita Universidad Autónoma de Puebla, 72570 Puebla, Mexico; b Instituto de Física, Benemérita Universidad Autónoma de Puebla, 72570 Puebla, Mexico; c Centro Universitario de Vinculación, Benemérita Universidad Autónoma de Puebla, 72570 Puebla, Pue., Mexico; Sunway University, Malaysia

**Keywords:** crystal structure, urethane, hydrogen bond, centrosymmetric monomer

## Abstract

The title compound, C_20_H_16_N_2_O_6_ (systematic name: 4-hy­droxy­phenyl *N*-{4-[(4-hy­droxy­phen­oxy­carbon­yl)amino]­phen­yl}carbamate), contains two urethane groups substituting the central benzene ring in *para* positions. The mol­ecule is centrosymmetric, and displays a twisted conformation for the three aromatic rings [the dihedral angle between central benzene ring and the urethane group is 33.4 (6)°, and that between the latter and the terminal ring is 65.1 (1)°]. In the crystal, a three-dimensional framework is formed through O—H⋯O and N—H⋯O hydrogen bonds involving the hy­droxy and urethane functional groups, respectively.

## Abstract 2

The crystal structure of the title compound features a three-dimensional framework resulting from hydrogen bonds formed by the hy­droxy and urethane groups.

## Graphical abstract

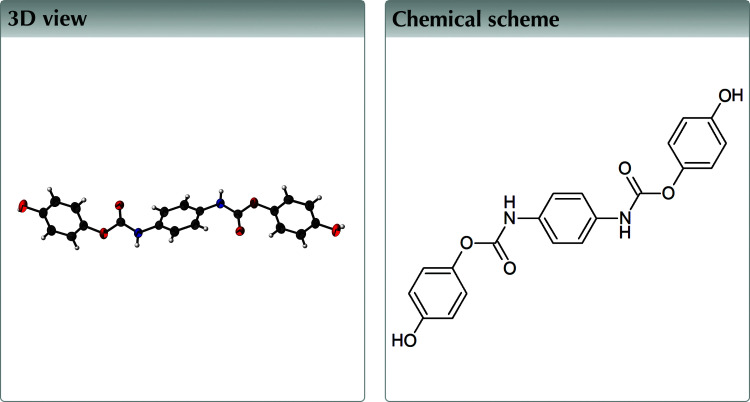

## Structure description

**Figure 1:**
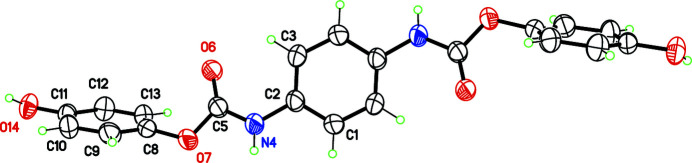
Mol­ecular structure of the title compound, with displacement ellipsoids shown at the 50% probability level. Non-labelled atoms are generated by symmetry operation 1 − *x*, −*y*, 1 − *z*.

The title compound was obtained by reacting hydro­quinone, 1,4-phenyl­ene diiso­cyanate and tri­ethyl­amine in dioxane. The resulting bis-urethane derivative crystallizes in the centrosymmetric space group *P*2_1_/*c*, with the mol­ecule having crystallographic inversion symmetry (Fig. 1 ▸). The urethane group displays the expected nearly planar geometry. This functional group is well represented in the CSD: 5700 hits are retrieved for organic compounds including an acyclic C—NH—(COO)—C fragment (CSD v. 5.43 with two updates, Groom *et al.*, 2016 ▸). However, most of these urethane derivatives originate from boc-protected amines, using the *tert*-but­oxy­carbonyl (boc) protecting group. In contrast, benzene rings substituted by two urethane groups are less studied by X-ray diffraction. For *para*-substituted benzene, only five structures have been deposited to date in the CSD. These occurrences include dimethyl 1,4-phenyl­enebiscarbamate (Stapf *et al.*, 2015 ▸), intended for anion complexation, and a dicholesterol derivative (Alegre-Requena *et al.*, 2020 ▸), intended for the preparation of supra­molecular gels.

As for dimethyl 1,4-phenyl­enebiscarbamate, the title mol­ecule is not planar. The dihedral angle between the central benzene ring and the urethane group is 33.4 (6)°, hindering the formation of an intra­molecular hydrogen bond C3—H3*A*⋯O6, although this could potentially stabilize the mol­ecule through the formation of an *S*(6) ring motif. The peripheral hy­droxy­benzene group is also rotated with respect to the urethane group, forming a dihedral angle of 65.1 (1)°.

**Figure 2:**
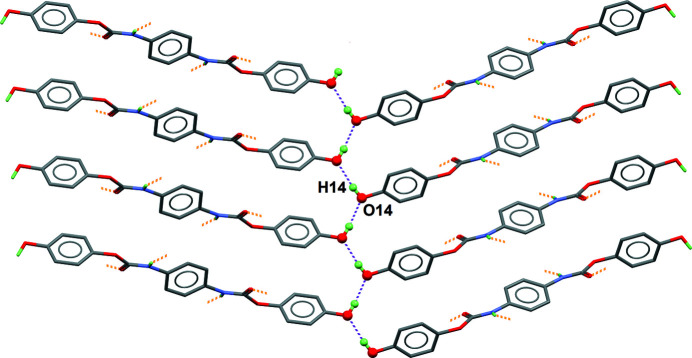
Supra­molecular layers formed by O—H⋯O hydrogen bonds (see entry 1 in Table 1 ▸). Hydrogen bonds are shown as purple dashed lines, and the projection is nearly normal to [001]. Note the hanging contacts (orange dashed lines), corresponding to the hydrogen bonds described in Fig. 3 ▸. Benzene-H atoms are omitted for clarity.

**Figure 3:**
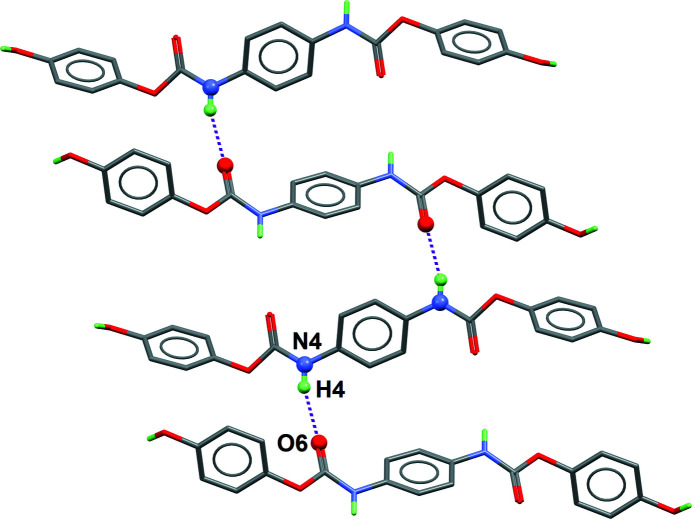
The two-dimensional supra­molecular motif formed by N—H⋯O hydrogen bonds (see entry 2 in Table 1 ▸). Two neighbouring mol­ecules are related by the glide plane of space group *P*2_1_/*c*. The projection is nearly normal to [010]. Benzene-H atoms are omitted for clarity.

This twisted mol­ecular conformation helps in the formation of two kinds of hydrogen bonds, leading to a three-dimensional supra­molecular architecture. First, hy­droxy groups behave both as donor and acceptor, linking mol­ecules through O—H⋯O hydrogen bonds. The resulting two-dimensional structure is nearly parallel to the (102) plane in the crystal (Table 1 ▸, entry 1; Fig. 2 ▸). These layers are further inter­connected by urethane N—H⋯O hydrogen bonds oriented nearly perpendicular to the layers (Table 1 ▸, entry 2; Fig. 3 ▸). The three-dimensional framework is thermodynamically stable, although no inter­molecular π–π inter­actions are present in the crystal.

The synthesized mol­ecule is a potential useful inter­mediate for obtaining other monomers, or cross-linking agents (Kothandaraman & Sultan Nasar, 1995 ▸; Lamba *et al.*, 1998 ▸): such diols are used for polycondensation reactions affording polymeric materials. On the other hand, some classes of urethane derivatives show diverse biological activity and have been used as fungicides, bactericides or analgesics, among other applications (Lamba *et al.*, 1998 ▸; Yagci *et al.*, 2011 ▸; Wang *et al.*, 2022 ▸).

## Synthesis and crystallization

The synthesis was performed in a 100 ml three-mouth flask, sealed with silicone grease and evacuated with argon. In 5 ml of dry dioxane, hydro­quinone (0.316 g), tri­ethyl­amine (0.207 ml) and 1,4-phenyl­ene diiso­cyanate (0.222 g) were added. The reaction was carried out at 353–363 K, under constant stirring. After a few minutes, it was observed that the reaction medium turned white. After 6 h, the reaction product was purified by column chromatography, using ethyl acetate:hexane (60:40) as the eluant. Once the purified monomer was obtained, it was dried in a furnace at 313 K for 24 h. Single crystals were obtained by evaporation of a saturated solution of the compound in an ethanol/di­chloro­methane mixture (4:1, *v*:*v*).

## Refinement

Crystal data, data collection and structure refinement details are summarized in Table 2 ▸.

## Supplementary Material

Crystal structure: contains datablock(s) I, global. DOI: 10.1107/S2414314622009191/tk4084sup1.cif

Structure factors: contains datablock(s) I. DOI: 10.1107/S2414314622009191/tk4084Isup2.hkl

Supporting information file. DOI: 10.1107/S2414314622009191/tk4084Isup3.cml

CCDC reference: 2207709

Additional supporting information:  crystallographic information; 3D view; checkCIF report

## Figures and Tables

**Table 1:** Hydrogen-bond geometry (Å, °)

*D*—H⋯*A*	*D*—H	H⋯*A*	*D*⋯*A*	*D*—H⋯*A*
O14—H14⋯O14^i^	0.85 (4)	1.91 (5)	2.754 (2)	172 (4)
N4—H4⋯O6^ii^	0.84 (3)	2.13 (4)	2.945 (3)	163 (3)

Symmetry codes: (i) 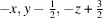 ; (ii) 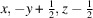 .

**Table 2:** Experimental details

Crystal data	
Chemical formula	C_20_H_16_N_2_O_6_
*M*_r_	380.35
Crystal system, space group	Monoclinic, *P*2_1_/*c*
Temperature (K)	295
*a*, *b*, *c* (Å)	19.0804 (17), 4.6758 (3), 10.1189 (8)
β (°)	101.169 (7)
*V* (Å^3^)	885.67 (12)
*Z*	2
Radiation type	Ag *K*α, λ = 0.56083 Å
μ (mm^−1^)	0.07
Crystal size (mm)	0.26 × 0.20 × 0.03

Data collection	
Diffractometer	Stoe Stadivari
Absorption correction	Multi-scan (*X-AREA*; Stoe & Cie, 2019 ▸)
*T*_min_, *T*_max_	0.450, 1.000
No. of measured, independent and observed [*I* > 2σ(*I*)] reflections	15028, 1678, 984
*R*_int_	0.074
(sin θ/λ)_max_ (Å^−1^)	0.609

Refinement	
*R*[*F* ^2^ > 2σ(*F* ^2^)], *wR*(*F* ^2^), *S*	0.051, 0.165, 1.06
No. of reflections	1678
No. of parameters	133
H-atom treatment	H atoms treated by a mixture of independent and constrained refinement
Δρ_max_, Δρ_min_ (e Å^−3^)	0.16, −0.20

Computer programs: *X-AREA* (Stoe & Cie, 2019 ▸), *SHELXT2018/2* (Sheldrick, 2015*a* ▸), *SHELXL2018/3* (Sheldrick, 2015*b* ▸), *XP* in *SHELXTL-Plus* (Sheldrick, 2008 ▸), *Mercury* (Macrae *et al.*, 2020 ▸) and *publCIF* (Westrip, 2010 ▸).

## full crystallographic data

### Crystal data

**Table:**

C_20_H_16_N_2_O_6_	*F*(000) = 396
*M**_r_* = 380.35	*D*_x_ = 1.426 Mg m^−^^3^
Monoclinic, *P*2_1_/*c*	Ag *K*α radiation, λ = 0.56083 Å
*a* = 19.0804 (17) Å	Cell parameters from 8468 reflections
*b* = 4.6758 (3) Å	θ = 2.6–25.0°
*c* = 10.1189 (8) Å	µ = 0.07 mm^−^^1^
β = 101.169 (7)°	*T* = 295 K
*V* = 885.67 (12) Å^3^	Plate, colourless
*Z* = 2	0.26 × 0.20 × 0.03 mm

### Data collection

**Table:**

Stoe Stadivari diffractometer	1678 independent reflections
Radiation source: Sealed X-ray tube, Axo Astix-f Microfocus source	984 reflections with *I* > 2σ(*I*)
Graded multilayer mirror monochromator	*R*_int_ = 0.074
Detector resolution: 5.81 pixels mm^-1^	θ_max_ = 20.0°, θ_min_ = 2.6°
ω scans	*h* = −23→23
Absorption correction: multi-scan (X-AREA; Stoe & Cie, 2019)	*k* = −5→5
*T*_min_ = 0.450, *T*_max_ = 1.000	*l* = −12→11
15028 measured reflections	

### Refinement

**Table:**

Refinement on *F*^2^	Primary atom site location: dual
Least-squares matrix: full	Secondary atom site location: difference Fourier map
*R*[*F*^2^ > 2σ(*F*^2^)] = 0.051	Hydrogen site location: mixed
*wR*(*F*^2^) = 0.165	H atoms treated by a mixture of independent and constrained refinement
*S* = 1.06	*w* = 1/[σ^2^(*F*_o_^2^) + (0.0826*P*)^2^] where *P* = (*F*_o_^2^ + 2*F*_c_^2^)/3
1678 reflections	(Δ/σ)_max_ < 0.001
133 parameters	Δρ_max_ = 0.16 e Å^−^^3^
0 restraints	Δρ_min_ = −0.19 e Å^−^^3^
0 constraints	

### Special details

**Table:**

Refinement. H atoms bonded to heteroatoms were refined freely, while H atoms of aromatic CH groups were placed in calculated positions and refined as riding to their carrier C atom.

### Fractional atomic coordinates and isotropic or equivalent isotropic displacement parameters (Å^2^)

**Table:**

	*x*	*y*	*z*	*U*_iso_*/*U*_eq_	
C1	0.47748 (17)	0.1997 (8)	0.4010 (3)	0.0515 (9)	
H1A	0.462350	0.335166	0.334332	0.062*	
C2	0.42963 (15)	0.0948 (7)	0.4754 (3)	0.0429 (8)	
C3	0.45239 (16)	−0.1062 (8)	0.5758 (3)	0.0476 (8)	
H3A	0.420723	−0.177251	0.626969	0.057*	
N4	0.35804 (14)	0.1974 (7)	0.4457 (3)	0.0519 (8)	
H4	0.3413 (19)	0.235 (8)	0.365 (4)	0.062*	
C5	0.31708 (16)	0.2435 (7)	0.5367 (3)	0.0431 (8)	
O6	0.33018 (11)	0.1848 (5)	0.65433 (18)	0.0521 (7)	
O7	0.25538 (11)	0.3727 (6)	0.47506 (19)	0.0613 (8)	
C8	0.20244 (17)	0.4238 (8)	0.5511 (3)	0.0462 (8)	
C9	0.13914 (17)	0.2829 (8)	0.5162 (3)	0.0487 (8)	
H9A	0.133664	0.144948	0.448748	0.058*	
C10	0.08313 (17)	0.3420 (7)	0.5796 (3)	0.0461 (8)	
H10A	0.039714	0.247497	0.554220	0.055*	
C11	0.09221 (16)	0.5420 (7)	0.6806 (3)	0.0398 (7)	
C12	0.15670 (17)	0.6839 (7)	0.7167 (3)	0.0479 (8)	
H12A	0.162645	0.819364	0.785254	0.058*	
C13	0.21180 (17)	0.6259 (8)	0.6520 (3)	0.0507 (9)	
H13A	0.255094	0.721890	0.675983	0.061*	
O14	0.03802 (12)	0.6086 (5)	0.7487 (2)	0.0505 (6)	
H14	0.011 (2)	0.464 (10)	0.749 (4)	0.076*	

### Atomic displacement parameters (Å^2^)

**Table:**

	*U*^11^	*U*^22^	*U*^33^	*U*^12^	*U*^13^	*U*^23^
C1	0.0432 (19)	0.073 (3)	0.0396 (15)	0.0162 (17)	0.0118 (14)	0.0108 (15)
C2	0.0348 (17)	0.060 (2)	0.0349 (14)	0.0080 (15)	0.0093 (13)	−0.0035 (14)
C3	0.0380 (18)	0.066 (2)	0.0420 (16)	0.0044 (16)	0.0162 (14)	0.0038 (15)
N4	0.0366 (16)	0.086 (2)	0.0351 (12)	0.0135 (14)	0.0118 (12)	0.0053 (14)
C5	0.0351 (17)	0.058 (2)	0.0359 (15)	0.0054 (15)	0.0059 (13)	−0.0010 (15)
O6	0.0419 (13)	0.0833 (19)	0.0320 (11)	0.0149 (12)	0.0097 (9)	0.0029 (10)
O7	0.0401 (13)	0.105 (2)	0.0415 (11)	0.0267 (13)	0.0142 (10)	0.0150 (12)
C8	0.0367 (18)	0.067 (2)	0.0364 (15)	0.0132 (16)	0.0117 (13)	0.0128 (15)
C9	0.0443 (19)	0.062 (2)	0.0386 (15)	0.0063 (16)	0.0056 (14)	−0.0052 (15)
C10	0.0347 (17)	0.054 (2)	0.0502 (17)	−0.0009 (15)	0.0100 (14)	−0.0042 (15)
C11	0.0373 (17)	0.0401 (19)	0.0446 (15)	0.0056 (14)	0.0145 (13)	0.0050 (14)
C12	0.047 (2)	0.047 (2)	0.0518 (18)	−0.0047 (16)	0.0141 (15)	−0.0109 (15)
C13	0.0343 (17)	0.064 (2)	0.0541 (18)	−0.0046 (16)	0.0092 (15)	0.0063 (17)
O14	0.0458 (13)	0.0474 (15)	0.0659 (14)	0.0011 (11)	0.0294 (11)	−0.0022 (11)

### Geometric parameters (Å, º)

**Table:**

C1—C2	1.381 (4)	C8—C13	1.377 (5)
C1—C3^i^	1.384 (4)	C9—C10	1.377 (4)
C1—H1A	0.9300	C9—H9A	0.9300
C2—C3	1.390 (5)	C10—C11	1.371 (4)
C2—N4	1.424 (4)	C10—H10A	0.9300
C3—H3A	0.9300	C11—C12	1.383 (4)
N4—C5	1.336 (4)	C11—O14	1.385 (3)
N4—H4	0.84 (3)	C12—C13	1.369 (4)
C5—O6	1.199 (3)	C12—H12A	0.9300
C5—O7	1.362 (4)	C13—H13A	0.9300
O7—C8	1.404 (3)	O14—H14	0.85 (4)
C8—C9	1.361 (5)		
			
C2—C1—C3^i^	121.0 (3)	C13—C8—O7	121.3 (3)
C2—C1—H1A	119.5	C8—C9—C10	120.8 (3)
C3^i^—C1—H1A	119.5	C8—C9—H9A	119.6
C1—C2—C3	119.5 (3)	C10—C9—H9A	119.6
C1—C2—N4	118.3 (3)	C11—C10—C9	119.1 (3)
C3—C2—N4	122.2 (3)	C11—C10—H10A	120.4
C1^i^—C3—C2	119.5 (3)	C9—C10—H10A	120.4
C1^i^—C3—H3A	120.3	C10—C11—C12	120.1 (3)
C2—C3—H3A	120.3	C10—C11—O14	121.7 (3)
C5—N4—C2	125.1 (3)	C12—C11—O14	118.2 (3)
C5—N4—H4	118 (2)	C13—C12—C11	120.3 (3)
C2—N4—H4	117 (2)	C13—C12—H12A	119.8
O6—C5—N4	127.6 (3)	C11—C12—H12A	119.8
O6—C5—O7	123.5 (3)	C12—C13—C8	119.3 (3)
N4—C5—O7	109.0 (2)	C12—C13—H13A	120.4
C5—O7—C8	118.3 (2)	C8—C13—H13A	120.4
C9—C8—C13	120.4 (3)	C11—O14—H14	110 (3)
C9—C8—O7	118.2 (3)		
			
C3^i^—C1—C2—C3	0.5 (6)	C5—O7—C8—C13	69.9 (4)
C3^i^—C1—C2—N4	−179.3 (3)	C13—C8—C9—C10	0.9 (5)
C1—C2—C3—C1^i^	−0.5 (6)	O7—C8—C9—C10	−174.5 (3)
N4—C2—C3—C1^i^	179.3 (3)	C8—C9—C10—C11	−1.2 (5)
C1—C2—N4—C5	−144.0 (3)	C9—C10—C11—C12	0.7 (5)
C3—C2—N4—C5	36.2 (5)	C9—C10—C11—O14	−179.4 (3)
C2—N4—C5—O6	−7.1 (6)	C10—C11—C12—C13	0.0 (5)
C2—N4—C5—O7	172.8 (3)	O14—C11—C12—C13	−179.8 (3)
O6—C5—O7—C8	−4.1 (5)	C11—C12—C13—C8	−0.3 (5)
N4—C5—O7—C8	175.9 (3)	C9—C8—C13—C12	−0.2 (5)
C5—O7—C8—C9	−114.7 (4)	O7—C8—C13—C12	175.1 (3)

Symmetry code: (i) −*x*+1, −*y*, −*z*+1.

### Hydrogen-bond geometry (Å, º)

**Table:**

*D*—H···*A*	*D*—H	H···*A*	*D*···*A*	*D*—H···*A*
C3—H3*A*···O6	0.93	2.47	2.939 (4)	111
C13—H13*A*···O6^ii^	0.93	2.63	3.451 (4)	148
O14—H14···O14^iii^	0.85 (4)	1.91 (5)	2.754 (2)	172 (4)
N4—H4···O6^iv^	0.84 (3)	2.13 (4)	2.945 (3)	163 (3)

Symmetry codes: (ii) *x*, *y*+1, *z*; (iii) −*x*, *y*−1/2, −*z*+3/2; (iv) *x*, −*y*+1/2, *z*−1/2.
